# Material-Oriented Shape Functions for FGM Plate Finite Element Formulation

**DOI:** 10.3390/ma13030803

**Published:** 2020-02-10

**Authors:** Wojciech Gilewski, Jan Pełczyński

**Affiliations:** Faculty of Civil Engineering, Warsaw University of Technology, 00-637 Warsaw, Poland; j.pelczynski@il.pw.edu.pl

**Keywords:** finite element, FGM, plate, material-oriented shape functions, NURBS

## Abstract

A four-noded finite element of a moderately thick plate made of functionally graded material (FGM) is presented. The base element is rectangular and can be extended to any shape using a transformation based on NURBS functions. The proposed 2D shape functions are consistent with the physical interpretation and describe the states of element displacement caused by unit displacements of nodes. These functions depend on the FGM’s material parameters and are called material-oriented. The shape function matrix is based on a superposition displacement field of two plate strips with 1D exact shape functions. A characteristic feature of the proposed formulation is full coupling of the membrane and bending states in the plate. The analytical form of the stiffness matrix and the nodal load vector was obtained, which leads to the numerical efficiency of the formulation. The element has been incorporated into Abaqus software with the use of Maple program. The finite element shows good convergence properties for different FGM models in the transverse direction to the middle plane of the plate. During derivation of the 2D plate element the formally exact 1D finite element for transverse nonhomogeneous FGM plate strip was developed.

## 1. Introduction

Functionally graded materials (FGMs) belong to a class of innovative materials with varying properties over a changing dimension [1,2]. The materials both occur in nature and can be obtained in an artificial way. A good example of an FGM found in nature is bamboo wood [1,2]. Due to the natural fiber distribution in the stem cross-section, it has high bending strength under natural loads. The beginnings of conscious creation of artificial FGMs should be dated to the beginning of the 1980s, with applications for the construction of thermal shields with unprecedented parameters. FGM eliminates the sharp interfaces existing in composite materials, replacing them with a gradient interface to produce a smooth transition from one material to the next.

There are different kinds of fabrication processes for producing functionally graded materials. Thin FGM sections are produced by physical or chemical vapor depositions, plasma spraying, self-propagating high temperature synthesis, etc. Volume FGM members are produced using powder metallurgy technique, centrifugal casting method, solid freedom fabrication technology, etc. Further details can be found in the literature [1,2].

Applications of functionally graded materials are quite wide, from aerospace, energy and automobile, through mechanical and civil engineering, to medicine, sport, sensors and optoelectronic fields. As the fabrication process is improved, the overall process cost is reduced, hence expanding the applications of FGM.

Plate structures are an important area of FGM applications. It is possible to build effective two-dimensional theories in which material variation in the transverse direction is usually assumed. Shear deformable as well as thin plate theories are to be applied. An overview of important papers in this field and the derivation of the equations of the first and higher order theories can be found in [3]. The scope of obtaining effective solutions by means of analytical methods is very limited (see [3]). It is necessary to use computational methods, among which the finite element method [4] dominates.

In the standard FEM model, the functional variability of material coefficients is represented in the elasticity matrix, which appears in the formulas for the finite element stiffness matrix. Shape functions are usually polynomial, with varying numbers of nodes and various techniques for improving formulation properties. A synthetic description of the most important models, together with citations of representative papers, is presented below.

Formulation within three-dimensional theory was applied to static analysis of thick or moderately thick rectangular plates [5] (eight-node brick element), to circular plates on elastic foundation [6] as well as for vibration analysis [7] (8- and 20-node isoparametric elements). Gradation of the finite elements’ elastic properties can be also used in the 3D formulation.

Various two-dimensional theories with the influence of shear deformation are the most common approach. Usually the first-order formulations are applied.

Rectangular finite elements with linear shape functions were used for static analysis, piezothermoelastic dynamics [8], active deflection/vibration control [9] and free vibrations [10]. Eight-node rectangular elements were applied for nonlinear free flexural vibrations [11] and piezo- electric fiber reinforcement [12]. The enhanced strain formulation to avoid the locking effects in eight-node elements was proposed in [13] for structural stability and dynamics problems. The elements can be successfully implemented in the Abaqus package [14].

Triangular plate finite elements with the discrete shear gap improvement were analyzed in [15,16] for static and free vibration problems.

Axi-symmetric plate finite elements within buckling, vibration and thermal analysis were proposed in [17].

Nine-node quadrilateral finite element was proposed for higher order shear deformation theory for static and free vibration analysis in [18] and was extended for large amplitude problems in [19]. Modified discrete Kirchhoff four-node quadrilateral element was used for the third-order theory for skew plates in [20].

An example of the application of C-1 continuity finite element formulated for classical thin FGM plate theory and applied for natural frequency analysis can be found in [21].

Isogeometric finite element formulation with NURBS-based shape functions was presented in [22] for static, vibrations, buckling and flutter as well as in [23] for thermal buckling.

The finite element formulation can be also applied for FGM shell structures and FGM beams—see [24,25,26,27,28] as representative papers.

A variety of finite element formulations applied for FGM plates seems to be a sign that the development of an element suitable for the analysis is still an open problem. This was an inspiration for the proposal of the present paper. The common problems with FE formulations based on standard polynomial shape functions are shear locking (connected to the parasitic shear) and zero energy modes. Analogous problems occur in FGM modeling using FEM. In addition, standard polynomial approximation assumes decoupling of approximated displacement fields. This is not consistent with the physical interpretation of the shape function in FGM boards, which should describe the displacement fields caused by unit displacement of nodes and should be coupled. In the matrices of shape functions proposed in the work, we observe full displacement of displacement fields.

The proposed elements are free from zero energy modes. Earlier studies of homogeneous plates with similar physical shape functions [29] did not show shear locking even for very thin plates. This phenomenon was also not observed in the FGM model proposed in this paper. The new finite elements are based on the physical interpretation of the shape functions, according to which they describe the distribution of finite element displacements caused by unit displacement of nodes. Naturally, such distributions should depend on the physical properties of the element. This approach was proposed for the statics of homogeneous plate bending in [30] and generalized in [29,31,32] for other types of structures. The proposed base element is rectangular with fully coupled displacement fields (membrane and bending conditions), which are based on analytical solutions regarding plate strips constructed of material with any FGM properties. The developed finite element was incorporated into the Abaqus system with UEL element user subroutine (using Maple program procedures) and its convergence was tested. The basic four-node rectangular finite element can be extended to any shape via parametric transformation based on NURBS functions [33]. During the development of the rectangular element, a formally accurate finite element for plate strip was created.

## 2. FGM plate Finite Elements

**Figure 1:**
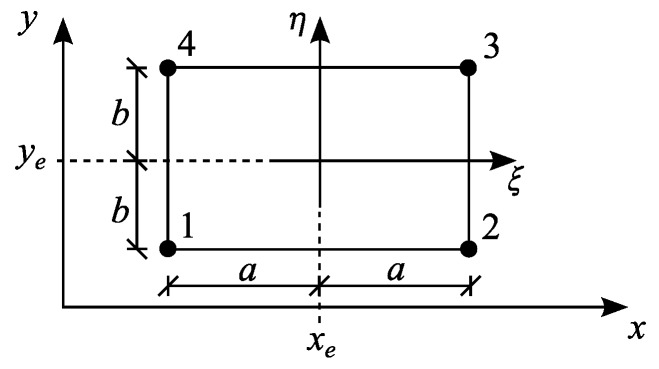
Four-noded rectangular plate element.

The subject under consideration is a rectangular four-noded finite element of dimensions 2*a* × 2*b* and thickness *h* in nondimensional coordinate system $\xi =\frac{x-x_{e}}{a},\eta =\frac{y-y_{e}}{b}$. The element is presented in Figure 1.

Generalized displacements of membrane and bending states as well as natural nodal displacements are defined as

(1) $$u=\{u,v,w,\varphi_{x},\varphi_{y}\}$$

(2) $$q=\{q_{1,}q_{2,}q_{3,}q_{4}\},$$

(3) $$q_{i}=\{u_{i},v_{i},\varphi_{xi},\varphi_{yi}\},\;i=1,2,3,4$$

Shape function matrix (u=Nq) is proposed in the form

(4) $$N=\left[N_{1}N_{2}N_{3}N_{4}\right],$$

(5) $$N_{i}=(\xi ,\eta )==[\begin{array}{ccccc} {\hat{N}}_{11}^{i}{\hat{\hat{N}}}_{11}^{i} & {\hat{N}}_{12}^{i}{\hat{\hat{N}}}_{22}^{i} & {\hat{N}}_{12}^{i}{\hat{\hat{N}}}_{22}^{i} & {\hat{N}}_{13}^{i}{\hat{\hat{N}}}_{22}^{i} & {\hat{N}}_{12}^{i}{\hat{\hat{N}}}_{23}^{i}\\ {\hat{N}}_{21}^{i}{\hat{\hat{N}}}_{12}^{i} & {\hat{N}}_{11}^{i}{\hat{\hat{N}}}_{11}^{i} & {\hat{N}}_{22}^{i}{\hat{\hat{N}}}_{12}^{i} & {\hat{N}}_{23}^{i}{\hat{\hat{N}}}_{12}^{i} & {\hat{N}}_{22}^{i}{\hat{\hat{N}}}_{13}^{i}\\ {\hat{N}}_{21}^{i}{\hat{\hat{N}}}_{22}^{i} & {\hat{N}}_{12}^{i}{\hat{\hat{N}}}_{21}^{i} & {\hat{N}}_{22}^{i}{\hat{\hat{N}}}_{22}^{i} & {\hat{N}}_{23}^{i}{\hat{\hat{N}}}_{22}^{i} & {\hat{N}}_{22}^{i}{\hat{\hat{N}}}_{23}^{i}\\ {\hat{N}}_{31}^{i}{\hat{\hat{N}}}_{22}^{i} & {\hat{N}}_{22}^{i}{\hat{\hat{N}}}_{21}^{i} & {\hat{N}}_{32}^{i}{\hat{\hat{N}}}_{22}^{i} & {\hat{N}}_{33}^{i}{\hat{\hat{N}}}_{22}^{i} & {\hat{N}}_{32}^{i}{\hat{\hat{N}}}_{23}^{i}\\ {\hat{N}}_{21}^{i}{\hat{\hat{N}}}_{32}^{i} & {\hat{N}}_{22}^{i}{\hat{\hat{N}}}_{31}^{i} & {\hat{N}}_{22}^{i}{\hat{\hat{N}}}_{32}^{i} & {\hat{N}}_{23}^{i}{\hat{\hat{N}}}_{32}^{i} & {\hat{N}}_{22}^{i}{\hat{\hat{N}}}_{33}^{i} \end{array}]$$

**Figure 2:**
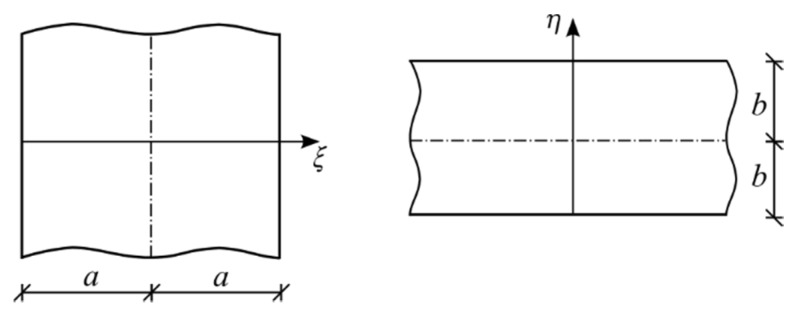
Crossed plate strips.

The matrix (Equation (5)) is consistent with physical interpretation of the shape function, according to which it describes the distribution of finite element displacements caused by unit displacement of nodes. This approach is an extension of the concept proposed for the statics of homogeneous plates [29] and for other types of structures [30,31]. Fully coupled displacement fields for membrane and bending states can be observed. The proposed shape matrix is an overlay of one-variable distributions of displacements of two crossed plate strips (Figure 2) with imposed boundary displacements.

(6) $$\hat{f}=\hat{f}(\xi ),\;\hat{\hat{f}}=\hat{\hat{f}}(\eta )$$

One-variable displacement fields are related to the FGM plate strips (Figure 2). If the first-order plate theory with shear deformation is used [3], the following six-level set of differential equations with physical boundary conditions is to be considered (for the *x* direction as the example):

(7) $$\left[\begin{array}{ccc} A_{0}\frac{1}{a^{2}}\frac{d^{2}}{d\xi^{2}} & 0 & -A_{1}\frac{1}{a^{2}}\frac{d^{2}}{d\xi^{2}}\\ 0 & -B_{0}\frac{1}{a^{2}}\frac{d^{2}}{d\xi^{2}} & B_{0}\frac{1}{a}\frac{d}{d\xi }\\ -A_{1}\frac{1}{a^{2}}\frac{d^{2}}{d\xi^{2}} & B_{0}\frac{1}{a}\frac{d}{d\xi } & A_{2}\frac{1}{a^{2}}\frac{d^{2}}{d\xi^{2}}-B_{0} \end{array}\right]\left[\begin{array}{c} u\\ w\\ \varphi \end{array}\right]=\left[\begin{array}{c} -p\\ -q\\ -m \end{array}\right]$$

where

(8) Ai=Eo1−v2∫−h2h2[P(z)⋅zi]dz, B0=561−ν2A0, i=0,1,2,

and *p, q* and *m* are load components. Coefficients $A_{i}$ and $B_{0}$ depend on the adopted FGM distribution represented for isotropic transverse nonhomogeneous material by the Young’s modulus $E_{0}$, Poisson’s ratio ν and function *P(z)*. In the above system of equations, one can observe a coupling of the membrane and bending states in the FGM panel. A similar system of equations for a moderately thick beam made of FGM was considered in the paper [26].

Solution of the homogeneous equations can be used to obtain exact shape functions for the strip, as follows:

(9) $$u\left(\xi \right)=C_{5}+C_{6}\xi +\frac{3A_{1}}{A_{0}a}C_{4}\xi^{2},$$

(10) $$w\left(\xi \right)=C_{1}+C_{2}\xi +C_{3}\xi^{2}+C_{4}\xi^{3},$$

(11) $$\phi \left(\xi \right)=\frac{1}{a}[C_{2}+2C_{3}\xi +3C_{4}(\xi^{2}+\frac{2A_{2}}{B_{0}a^{2}}-\frac{2A_{1}^{2}}{A_{0}B_{0}a^{2}})]$$

Two 1D displacement vectors $\hat{u}=\left(\xi \right)$ and $\hat{\hat{u}}=\left(\eta \right)$ for plate strips (Figure 2) are defined as

(12) $$\hat{u}=\left(\xi \right)=\{u(\xi ),w(\xi ),\varphi_{x}(\xi )\},\;\hat{\hat{u}}=\left(\eta \right)=\{v(\eta ),w(\eta ),\varphi_{y}(\eta )\}$$

Formally exact shape function matrices can be developed for each plate strip:

(13) $$\hat{u}=\left(\xi \right)=\hat{N}\left(\xi \right)\cdot {\hat{q}}_{e},\;\hat{\hat{u}}=\left(\eta \right)=\hat{\hat{N}}\left(\eta \right)\cdot {\hat{\hat{q}}}_{e}$$

with natural boundary parameters at each end:

(14) $${\hat{q}}_{e}=\{u_{1},w_{1},\varphi_{x1},u_{2},w_{2},\varphi_{x2}\},\;{\hat{\hat{q}}}_{e}=\{v_{1},w_{1},\varphi_{y1},v_{2},w_{2},\varphi_{y2}\}$$

The 1D shape function matrices can be expressed in the form

(15) $$\hat{N}=\left[\begin{array}{cc} {\hat{N}}_{1}\left(\xi \right) & {\hat{N}}_{2}\left(\xi \right) \end{array}\right],\;\hat{\hat{N}}=\left[\begin{array}{cc} {\hat{\hat{N}}}_{1}\left(\eta \right) & {\hat{\hat{N}}}_{2}\left(\eta \right) \end{array}\right]$$

(16) $${\hat{N}}^{i}\left(\xi \right)=\left[\begin{array}{ccc} {\hat{N}}_{11}^{i}\left(\xi \right) & {\hat{N}}_{12}^{i}\left(\xi \right) & {\hat{N}}_{13}^{i}\left(\xi \right)\\ 0 & {\hat{N}}_{22}^{i}\left(\xi \right) & {\hat{N}}_{23}^{i}\left(\xi \right)\\ 0 & {\hat{N}}_{32}^{i}\left(\xi \right) & {\hat{N}}_{33}^{i}\left(\xi \right) \end{array}\right],\;{\hat{\hat{N}}}^{i}\left(\eta \right)=\left[\begin{array}{ccc} {\hat{\hat{N}}}_{11}^{i}\left(\eta \right) & {\hat{\hat{N}}}_{12}^{i}\left(\eta \right) & {\hat{\hat{N}}}_{13}^{i}\left(\eta \right)\\ 0 & {\hat{\hat{N}}}_{22}^{i}\left(\eta \right) & {\hat{\hat{N}}}_{23}^{i}\left(\eta \right)\\ 0 & {\hat{\hat{N}}}_{32}^{i}\left(\eta \right) & {\hat{\hat{N}}}_{33}^{i}\left(\eta \right) \end{array}\right]$$

where

(17) $${\hat{N}}_{11}^{i}(\xi )=\frac{1}{2}\left(1+\xi_{i}\xi \right),\;{\hat{N}}_{12}^{i}(\xi )=\frac{3}{4}\frac{\xi_{i}A_{1}B_{0}a\left(1-\xi^{2}\right)}{3A_{0}A_{2}+A_{0}B_{0}a^{2}-3A_{1}^{2}},\;{\hat{N}}_{13}^{i}(\xi )=\frac{3}{4}\frac{A_{1}B_{0}a^{2}(\xi^{2}-1)}{3A_{0}A_{2}+A_{0}B_{0}a^{2}-3A_{1}^{2}},\;{\hat{N}}_{22}^{i}(\xi )=\frac{1}{2}+\frac{1}{2}\left(1+\frac{1}{2}\frac{A_{0}B_{0}a^{2}\left(1-\xi^{2}\right)}{3A_{0}A_{2}+A_{0}B_{0}a^{2}-3A_{1}^{2}}\right)\xi_{i}\xi ,\;{\hat{N}}_{23}^{i}(\xi )=\frac{1}{4}\left(\frac{A_{0}B_{0}a^{3}\xi }{3A_{0}A_{2}+A_{0}B_{0}a^{2}-3A_{1}^{2}}+a\xi_{i}\right)(\xi^{2}-1),\;{\hat{N}}_{32}^{i}(\xi )=\frac{3}{4}\frac{\xi_{i}A_{0}B_{0}a(1-\xi^{2})}{3A_{0}A_{2}+A_{0}B_{0}a^{2}-3A_{1}^{2}}\;{\hat{N}}_{33}^{i}(\xi )=\frac{1}{2}\left(\xi_{i}\xi +\frac{3}{2}\frac{A_{0}B_{0}a^{2}(\xi^{2}-1)}{3A_{0}A_{2}+A_{0}B_{0}a^{2}-3A_{1}^{2}}+1\right).$$

The shape functions for the second direction are analogous. They can be obtained from Equation (17) by exchanging variables ξ→η,a→b. The functions (Equation (17)) strongly depend on the parameters of FGM, so the formulation is named “material-oriented”.

Following the above formulation, the formally exact stiffness matrix as well as the load vector for two line-noded, 6 d.o.f. (horizontal displacements, vertical displacements and rotations) plate strip finite element can be expressed in the form (for the ξ direction as an example)

(18) $$K_{e}=\left[\begin{array}{cccccc} k_{11} & 0 & k_{13} & -k_{11} & 0 & -k_{13}\\ 0 & k_{22} & ak_{22} & 0 & -k_{22} & ak_{22}\\ k_{13} & ak_{22} & k_{33} & -k_{13} & -ak_{22} & k_{36}\\ -k_{11} & 0 & -k_{13} & k_{11} & 0 & k_{13}\\ 0 & -k_{22} & -ak_{22} & 0 & k_{22} & -ak_{22}\\ -k_{13} & ak_{22} & k_{36} & k_{13} & -ak_{22} & k_{33} \end{array}\right],$$

(19) $$k_{11}=\frac{A_{0}}{2a},\;k_{13}=-\frac{A_{1}}{2a},\;k_{22}=\frac{3B_{0}\left(A_{0}A_{2}-A_{1}^{2}\right)}{2a\left[-3\left(A_{1}^{2}-A_{0}A_{2}\right)+a^{2}A_{0}B_{0}\right]},\;k_{33}=\frac{-3A_{1}^{2}\left(A_{2}+a^{2}B_{0}\right)+A_{0}A_{2}\left(3A_{2}+4a^{2}B_{0}\right)}{2a\left[-3\left(A_{1}^{2}-A_{0}A_{2}\right)+a^{2}A_{0}B_{0}\right]},\;k_{36}=\frac{3A_{1}^{2}\left(A_{2}-a^{2}B_{0}\right)-A_{0}A_{2}\left(3A_{2}-2a^{2}B_{0}\right)}{2a\left[-3\left(A_{1}^{2}-A_{0}A_{2}\right)+a^{2}A_{0}B_{0}\right]},$$

(20) $$Q_{e}=pa[\begin{array}{c} 1\\ -\frac{aA_{1}B_{0}}{-3A_{1}^{2}+A_{0}+(3A_{2}+a^{2}B_{0})}\\ -\frac{a^{2}A_{1}B_{0}}{-3A_{1}^{2}+A_{0}+(3A_{2}+a^{2}B_{0})}\\ 1\\ -\frac{aA_{1}B_{0}}{-3A_{1}^{2}+A_{0}+(3A_{2}+a^{2}B_{0})}\\ -\frac{a^{2}A_{1}B_{0}}{-3A_{1}^{2}+A_{0}+(3A_{2}+a^{2}B_{0})} \end{array}]+qa[\begin{array}{c} 0\\ 1\\ \frac{1}{3}a\\ 0\\ 1\\ -\frac{1}{3}a \end{array}]+ma[\begin{array}{c} 0\\ -\frac{aA_{0}B_{0}}{-3A_{1}^{2}+A_{0}+(3A_{2}+a^{2}B_{0})}\\ -\frac{-3A_{1}^{2}+3A_{1}A_{2}}{-3A_{1}^{2}+A_{0}+(3A_{2}+a^{2}B_{0})}\\ 0\\ -\frac{aA_{0}B_{0}}{-3A_{1}^{2}+A_{0}+(3A_{2}+a^{2}B_{0})}\\ -\frac{-3A_{1}^{2}+3A_{1}A_{2}}{-3A_{1}^{2}+A_{0}+(3A_{2}+a^{2}B_{0})} \end{array}]$$

The results to be obtained within the above formulation are formally exact and no convergence study is necessary.

The strain matrix for the rectangular finite element can be expressed as

(21) $$B=\left[B_{1}B_{2}B_{3}B_{4}\right],\;\;B_{i}=DN_{i},$$

where

(22) $$D=[\begin{array}{cc} D_{1} & 0\\ 0 & D_{2} \end{array}],\;D_{1}=[\begin{array}{cc} \frac{1}{a}\frac{\partial }{\partial \xi } & 0\\ 0 & \frac{1}{b}\frac{\partial }{\partial \eta }\\ \frac{1}{b}\frac{\partial }{\partial \eta } & \frac{1}{a}\frac{\partial }{\partial \xi } \end{array}],\;D_{2}=[\begin{array}{ccc} 0 & -\frac{1}{a}\frac{\partial }{\partial \xi } & 0\\ 0 & 0 & -\frac{1}{b}\frac{\partial }{\partial \eta }\\ 0 & -\frac{1}{b}\frac{\partial }{\partial \eta } & -\frac{1}{a}\frac{\partial }{\partial \xi }\\ \frac{1}{a}\frac{\partial }{\partial \xi } & -1 & 0\\ \frac{1}{b}\frac{\partial }{\partial \eta } & 0 & -1 \end{array}]$$

The stiffness matrix and load vector can be received in the standard FEM procedure as follows:

(23) $$K_{e}=ab\int_{-1}^{1}\int_{-1}^{1}B^{T}EBd\xi d\eta ,$$

(24) $$Q_{e}=ab\int_{-1}^{1}\int_{-1}^{1}N^{T}pd\xi d\eta ,$$

where elasticity matrix is expressed as

(25) $$E=[\begin{array}{ccc} E_{1} & E_{2} & 0\\ E_{2}^{T} & E_{3} & 0\\ 0 & 0 & E_{4} \end{array}]$$

(26) $$E_{1}=[\begin{array}{ccc} A_{0} & vA_{0} & 0\\ vA_{0} & A_{0} & 0\\ 0 & 0 & \frac{1-v}{2}A_{0} \end{array}],\;E_{2}=[\begin{array}{ccc} A_{1} & vA_{1} & 0\\ vA_{1} & A_{1} & 0\\ 0 & 0 & 0 \end{array}],$$

(27) $$E_{3}=\left[\begin{array}{ccc} A_{2} & \nu A_{2} & 0\\ \nu A_{2} & A_{2} & 0\\ 0 & 0 & \frac{1-\nu }{2}A_{2} \end{array}\right],\;E_{4}=\left[\begin{array}{cc} B_{0} & 0\\ 0 & B_{0} \end{array}\right],$$

and

(28) $$p=\left\{p_{x},p_{y},p_{z},m_{x},m_{y}\right\}$$

is the load vector for the panel.

The analytical form of stiffness matrix can be obtained with the use of *Maple* software—the expressions are relatively long and exceed the limitations of the present paper.

The analytical form of the load vector can be expressed as

(29) $$Q_{e}=[\begin{array}{c} Q_{1}^{e}\\ Q_{2}^{e}\\ Q_{3}^{e}\\ Q_{4}^{e} \end{array}],$$

(30) $$\begin{array}{l} Q_{i}^{e}=p_{x}ab\left[\begin{array}{c} 1\\ 0\\ \frac{aA_{1}B_{0}}{3A_{0}A_{2}+A_{0}B_{0}a^{2}-3A_{1}^{2}}\xi_{i}\\ -\frac{a^{2}A_{1}B_{0}}{3A_{0}A_{2}+A_{0}B_{0}a^{2}-3A_{1}^{2}}\\ -\frac{1}{3}\frac{abA_{1}B_{0}}{3A_{0}A_{2}+A_{0}B_{0}a^{2}-3A_{1}^{2}}\xi_{i}\eta_{i} \end{array}\right]+p_{y}ab\left[\begin{array}{c} 0\\ 1\\ \frac{bA_{1}B_{0}}{3A_{0}A_{2}+A_{0}B_{0}a^{2}-3A_{1}^{2}}\eta_{i}\\ -\frac{1}{3}\frac{abA_{1}B_{0}}{3A_{0}A_{2}+A_{0}B_{0}a^{2}-3A_{1}^{2}}\xi_{i}\eta_{i}\\ -\frac{a^{2}A_{1}B_{0}}{3A_{0}A_{2}+A_{0}B_{0}a^{2}-3A_{1}^{2}} \end{array}\right]+p_{z}ab\left[\begin{array}{c} 0\\ 0\\ 1\\ -\frac{a\xi_{i}}{3}\\ -\frac{b\eta_{i}}{3} \end{array}\right]+\\ +m_{x}ab\left[\begin{array}{c} 0\\ 0\\ \frac{aA_{0}B_{0}}{3A_{0}A_{2}+A_{0}B_{0}a^{2}-3A_{1}^{2}}\xi_{i}\\ -\frac{a^{2}A_{0}B_{0}}{3A_{0}A_{2}+A_{0}B_{0}a^{2}-3A_{1}^{2}}\\ -\frac{1}{3}\frac{abA_{0}B_{0}}{3A_{0}A_{2}+A_{0}B_{0}a^{2}-3A_{1}^{2}}\xi_{i}\eta_{i} \end{array}\right]+m_{y}ab\left[\begin{array}{c} 0\\ 0\\ \frac{bA_{1}B_{0}}{3A_{0}A_{2}+A_{0}B_{0}a^{2}-3A_{1}^{2}}\eta_{i}\\ -\frac{1}{3}\frac{abA_{1}B_{0}}{3A_{0}A_{2}+A_{0}B_{0}a^{2}-3A_{1}^{2}}\xi_{i}\eta_{i}\\ -\frac{a^{2}A_{1}B_{0}}{3A_{0}A_{2}+A_{0}B_{0}a^{2}-3A_{1}^{2}} \end{array}\right]. \end{array}$$

The rectangular finite element with material-oriented shape functions satisfy rigid body motion, constant strain and ellipticity conditions [4,30].

The proposed finite element was introduced to the Abaqus system using the UEL element user subroutine (see [34,35] for details). The UEL procedure is written in FORTRAN. The stiffness matrix is entered into the code from Maple Software for Mathematica using the command Fortran (K, optimize, resultname = “AMATRX”) [36] using Equations (18–20) or (21–30).

For the purpose of the present paper, the analysis is limited to the isotropic plates with functional transverse gradation of Young’s modulus ($E,E_{0}$) and mass density ($\rho ,\rho_{0}$):

(31) $$E(z)=E_{0}\cdot P(z),v=const.,\;\rho (z)=\rho_{0}\cdot P(z).$$

Three FGM distributions (Equations (32–34)) were taken into consideration:

(32) $$P(z)=1+4\frac{z^{2}}{h^{2}}\gamma ,$$

(33) $$P(z)=(\eta -1)(\frac{z}{h}+\frac{1}{2}{)}^{n}+1,\;\eta =\frac{P_{t}}{P_{b}},$$

(34) $$P(z)=\frac{\delta /\pi }{\delta^{2}+{\left(\frac{z}{h}-\xi_{0}\right)}^{2}}+1,$$

where $\gamma {,}^{}P_{t}{,}^{}P_{b}{,}^{}n{,}^{}\delta {,}^{}\xi_{0}$ are constant parameters to model various FGM distributions. Here, “*t*” represents the top of the plate, “*b*” represents the bottom of the plate, $E_{0}=E_{b}{,}_{}\rho_{0}=\rho_{b}$.

**Figure 3:**
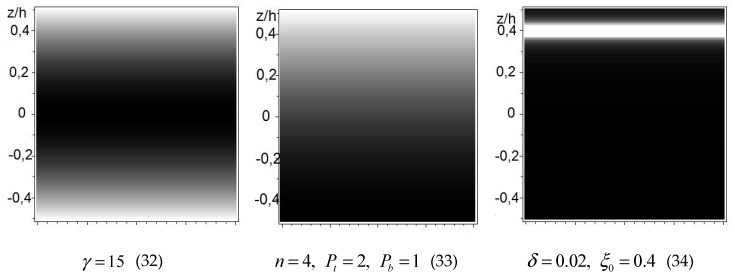
Selected transverse nonhomogeneous materials of the plate.

Examples of functions *P(z)* along thickness of the plate are presented in Figure 3.

Light color represents material with a higher intensity of material properties.

The functions described by Equations (32) and (33) are normally used to describe FGMs. The function described by Equation (34) is original and can be used to describe plates with reinforced mats glued in.

## 3. Numerical Examples

**Figure 4:**
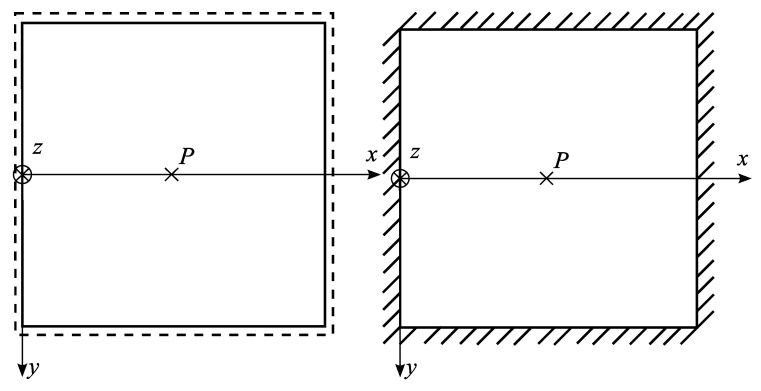
Plates taken for convergence analysis: simply supported and clamped with concentrated load in the middle.

The proposed finite element was adapted to the Abaqus software. To verify the finite element, several examples were made. Some of the examples made for convergence check are presented below. Square plates are considered (simply supported and clamped—Figure 4) with the following data: dimensions L×L, thickness h=L/10, Young’s modulus $E_{0}$, Poisson’s ratio ν=0.25, concentrated force P.

**Figure 5:**
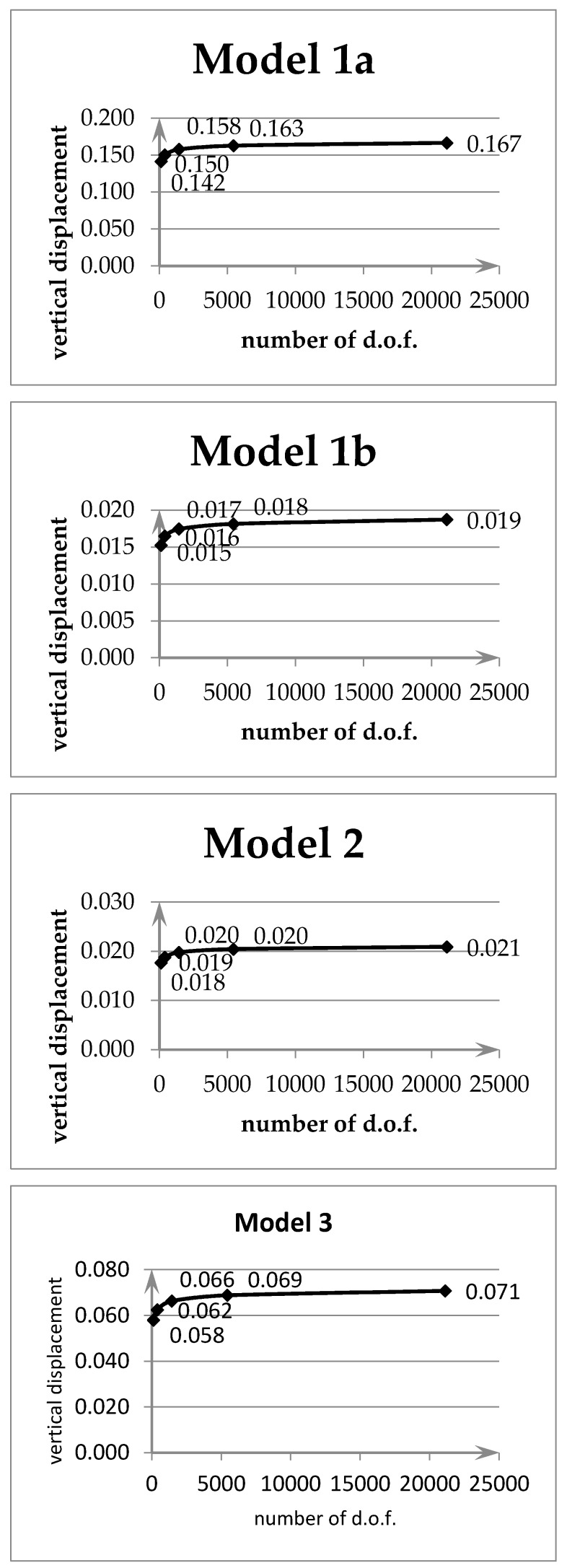
Convergence graphs for simply supported plate (multiplier $\frac{PL^{2}}{E_{0}h^{3}}$).

**Figure 6:**
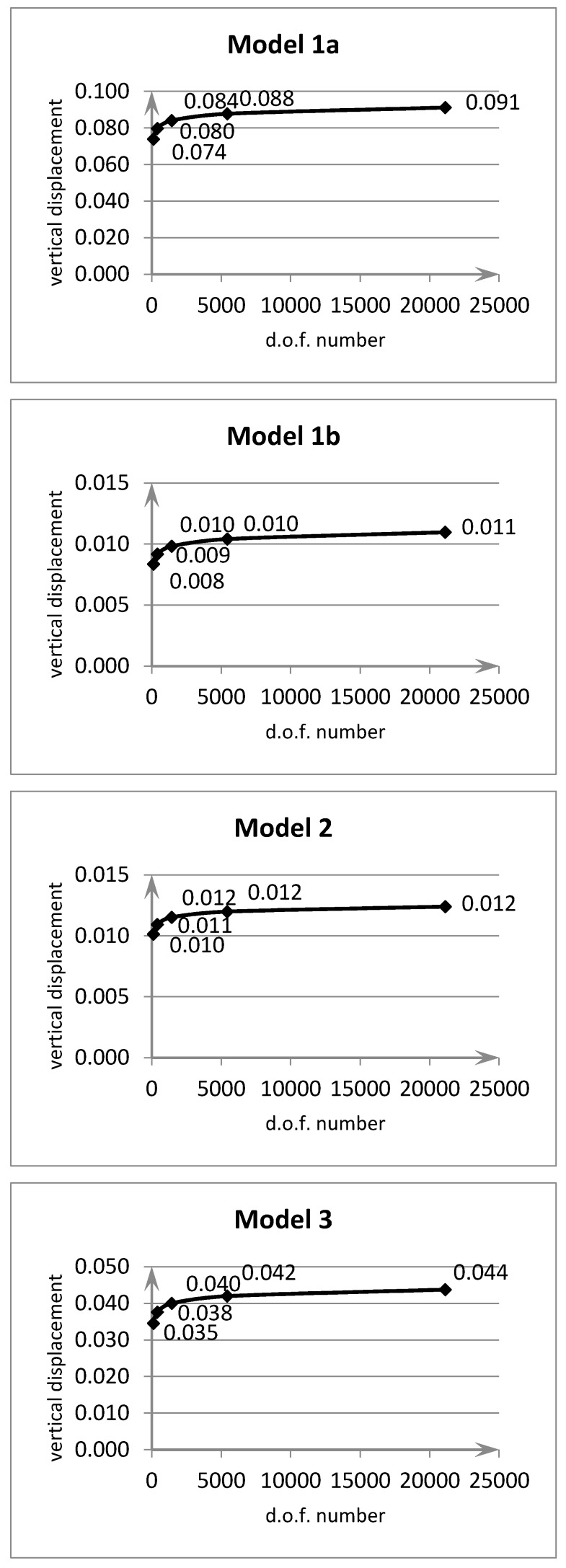
Convergence graphs for clamped plate (multiplier $\frac{PL^{2}}{E_{0}h^{3}}$).

In Figure 5 and Figure 6 convergence results are shown for the maximum vertical deflection in the middle of the plates.

Model 1a is a transverse homogeneous plate (for γ=0 according to Equation (32)). Model 1b is a transverse inhomogeneous plate according to Equation (32) for γ=15. Model 2 is a transverse inhomogeneous plate according to Equation (33) for $n=4{,}^{}P_{t}=P_{b}$. Model 3 is a transverse inhomogeneous plate according to Equation (34) for $\delta =0.02{,}^{}\xi_{0}=0.4$.

One can observe that each example is characterized by a good rate of convergence for all models.

The influence of the FGM model applied in comparison to the homogeneous plate is visible. The considerable differences result from the adopted calculation parameters in the functions described by Equations (32)–(34). These parameters describe strong material variation—hence the differences compared to a homogeneous material. The rectangular element shows correct convergence.

Validation of the proposed model in the scope of FGM was carried out by comparison with the analytical solution available in the literature—the benchmark presented in the [37] was selected. A comparison regarding the maximum displacement of a simply supported plate subjected to uniform loading for the material model (Equation (33)) is shown in Table 1.

The numerical solution for homogeneous plates (*k =* 0 and *k =* infinity) is consistent with the analytical solution [37] in the range of five significant digits. For other parameters of *n*, the proposed finite element gives slightly less than analytical results at a level of 1%–3% error.

It is worth noting that the solution in the field of cylindrical bending using Equations (18) and (20) is formally accurate for any FGM.

## 4. Free-Form Plate Finite Element—the Use of NURBS Functions

**Figure 7:**
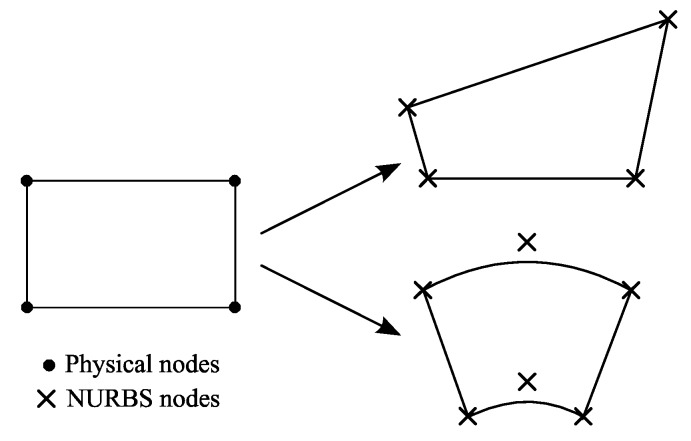
Example of free-form element shapes.

An extension of the proposed finite element for unrestricted shape (Figure 7—examples with four and six nodes) can be done by coordinate system transformation with the use of NURBS functions [33]:

(35) $$x={\sum_{i=1}^{N}F_{i}{\left(\xi ,\eta \right)}^{}x_{i},}^{}y=\sum_{i=1}^{N}F_{i}{\left(\xi ,\eta \right)}^{}y_{i},$$

where $F_{i}\left(\xi ,\eta \right)$, *i* = 1, …, *N*, are NURBS functions.

The formulation within description of geometry is close to superparametric [4]. Only four nodes are used in every element for representation of displacement fields (according to Equation (5)), as well as *N* nodes for description of a free-form element geometry.

The Jacobi and inverse Jacobi matrices can be obtained:

(36) $$J=\left[\begin{array}{cc} \frac{\partial x}{\partial \xi } & \frac{\partial y}{\partial \xi }\\ \frac{\partial x}{\partial \eta } & \frac{\partial y}{\partial \eta } \end{array}\right]=\left[\begin{array}{cc} \sum_{i=1}^{N}\frac{\partial F_{i}}{\partial \xi }x_{i} & \sum_{i=1}^{N}\frac{\partial F_{i}}{\partial \xi }y_{i}\\ \sum_{i=1}^{N}\frac{\partial F_{i}}{\partial \eta }x_{i} & \sum_{i=1}^{N}\frac{\partial F_{i}}{\partial \eta }y_{i} \end{array}\right],$$

(37) $$J^{-1}=\left[\begin{array}{cc} \frac{\partial \xi }{\partial x} & \frac{\partial \eta }{\partial x}\\ \frac{\partial \xi }{\partial y} & \frac{\partial \eta }{\partial y} \end{array}\right]=\frac{1}{J}\left[\begin{array}{cc} \frac{\partial y}{\partial \eta } & -\frac{\partial y}{\partial \xi }\\ -\frac{\partial x}{\partial \eta } & \frac{\partial x}{\partial \xi } \end{array}\right]=\left[\begin{array}{cc} J_{11}^{-1} & J_{12}^{-1}\\ J_{21}^{-1} & J_{22}^{-1} \end{array}\right].\;$$

where *J* is the Jacobian of this transformation

(38) $$J=\frac{\partial x}{\partial \xi }\frac{\partial y}{\partial \eta }-\frac{\partial x}{\partial \eta }\frac{\partial y}{\partial \xi }$$

Now, one can calculate derivatives necessary for development of the strain matrix:

(39) $$\frac{\partial \left(\ldots \right)}{\partial x}=\frac{\partial \left(\ldots \right)}{\partial \xi }J_{11}^{-1}+\frac{\partial \left(\ldots \right)}{\partial \eta }J_{12}^{-1},\;\frac{\partial \left(\ldots \right)}{\partial y}=\frac{\partial \left(\ldots \right)}{\partial \xi }J_{21}^{-1}+\frac{\partial \left(\ldots \right)}{\partial \eta }J_{22}^{-1},$$

Parameters *a* and *b*, necessary for the strain matrix, are average values of the element dimensions.

The strain matrix can be expressed in the form

(40) $$B=[\begin{array}{cccc} B_{1} & B_{2} & B_{3} & B_{4} \end{array}],\;B_{i}=DN_{i}$$

The above formulae provide a way to calculate the stiffness matrix and the load vector of an element:

(41) $$K^{e}=\underset{F^{e}}{\iint }B^{T}EB^{}dF^{e}=\overset{1}{\underset{-1}{\int }}\overset{1}{\underset{-1}{\int }}B^{T}\left(\xi ,\eta \right)EB{\left(\xi ,\eta \right)}^{}J^{}\;d\xi d\eta ,$$

(42) $$Q^{e}=\underset{F^{e}}{\iint }N^{T}P^{}dF^{e}=\overset{1}{\underset{-1}{\int }}\overset{1}{\underset{-1}{\int }}N^{T}PJ\;d\xi d\eta ,$$

where **E** is the elasticity matrix and **P** is the external load vector.

Following the above procedure, the limitation of the rectangular shape of the proposed finite element can be avoided.

## 5. Conclusions

The paper proposes a four-noded finite element of a moderately thick plate made of FGM. The base element is rectangular and can be extended to any shape using a transformation based on NURBS functions.

The shape functions in the considered elements are consistent with the physical interpretation and describe the states of element displacement caused by unit displacements of nodes. These functions depend on the FGM’s material parameters and are called material-oriented. A characteristic feature of the proposed formulation is full coupling of the membrane and bending states in the plate and the dependence of the shape functions on the FGM model.

The finite element meets the conditions of rigid body motion and constant strain as well as the elliptical condition. No spurious zero-energy modes were identified.

The analytical form of the stiffness matrix and the nodal load vector was obtained, which leads to the numerical efficiency of the formulation. The element has been incorporated into Abaqus software with the use of Maple program.

The finite element with material-oriented shape functions shows good convergence properties for different FGM models in the transverse direction to the middle plane of the plate. The element is free from locking effects. Examples have been completed to compare the FEM solution with the analytical solution. Basic examples were used to demonstrate the convergence and correctness of the proposed model. The results are promising as a base for modeling more complex plates built of FGMs with various distribution of the properties over the thickness.

During derivation of the 2D plate element, the formally exact 1D finite element for a transverse nonhomogeneous FGM plate strip was developed.

## Figures and Tables

**Table 1:** Comparison of the FEM results with analytical solutions [37] for a simply supported square plate (*L × L*, *h/L = 1/10*) with uniformly distributed load *q.* Material model (Equation (33)), $E_{t}=380GPa{,}^{}E_{b}=70GPa$ .

	$\tilde{w}=10\frac{E_{t}h^{3}}{qL^{4}}w(\frac{A}{2},\frac{B}{2})$						
*n*	0	1	2	3	5	10	Infinity
Analytical solution [37]	0.4665	0.9421	1.2228	1.3530	1.4647	1.6054	2.5328
Present study	0.4665	0.9280	1.1903	1.3124	1.4202	1.5692	2.5328
